# Recognition of Strokes in the ICU: A Narrative Review

**DOI:** 10.3390/jcdd10040182

**Published:** 2023-04-21

**Authors:** Kotaro Noda, Masatoshi Koga, Kazunori Toyoda

**Affiliations:** 1 Department of Cerebrovascular Medicine, National Cerebral and Cardiovascular Center, Suita 564-8565, Japan; 2 Department of Neurology and Neurological Science, Graduate School of Medical and Dental Sciences, Tokyo Medical and Dental University, Tokyo 113-8519, Japan

**Keywords:** in-hospital stroke, intensive care unit (ICU), perioperative stroke

## Abstract

Despite the remarkable progress in acute treatment for stroke, in-hospital stroke is still devastating. The mortality and neurological sequelae are worse in patients with in-hospital stroke than in those with community-onset stroke. The leading cause of this tragic situation is the delay in emergent treatment. To achieve better outcomes, early stroke recognition and immediate treatment are crucial. In general, in-hospital stroke is initially witnessed by non-neurologists, but it is sometimes challenging for non-neurologists to diagnose a patient’s state as a stroke and respond quickly. Therefore, understanding the risk and characteristics of in-hospital stroke would be helpful for early recognition. First, we need to know “the epicenter of in-hospital stroke”. Critically ill patients and patients who undergo surgery or procedures are admitted to the intensive care unit, and they are potentially at high risk for stroke. Moreover, since they are often sedated and intubated, evaluating their neurological status concisely is difficult. The limited evidence demonstrated that the intensive care unit is the most common place for in-hospital strokes. This paper presents a review of the literature and clarifies the causes and risks of stroke in the intensive care unit.

## 1. Introduction

In this decade, treatments for acute ischemic stroke (AIS) are developing radically. Intravenous (IV) thrombolysis is an effective treatment for AIS, and it is highly beneficial for patients with AIS up to 4.5 h after symptom onset [1,2]. Furthermore, Guidelines for the Early Management of Patients With Acute Ischemic Stroke recommended that even when the time of symptom onset is unclear or already over 4.5 h from the last known well, patients with AIS could receive IV thrombolysis if they showed diffusion-positive fluid-attenuated inversion recovery-negative lesions on magnetic resonance imaging (MRI) testing or perfusion mismatch on computed tomography (CT) or MRI perfusion [3]. In addition, randomized, controlled trials have proven the utility of endovascular thrombectomy (EVT) for AIS. Within 6 h from symptom onset, patients with AIS due to anterior large vessel occlusion have an opportunity to receive a beneficial effect from EVT. In addition, when a sufficient clinical-core mismatch is detected by computed tomography or MRI, and the patient fulfills the eligibility criteria of the DAWN trial or the DEFUSE-3 trial, the patients could be rescued by acute therapy even up to 24 h after onset [4,5].

Recently, the Recovery by Endovascular Salvage for Cerebral Ultra Acute Embolism Japan Large Ischemic Core Trial (RESCUE-Japan LIMIT), a randomized, multicenter study in Japan, showed that patients with a large ischemic core could enjoy better functional outcomes with EVT than with medical treatment alone [6]. Owing to those recent guidelines and studies, patients have a better chance of recovering from severe symptoms of AIS.

Despite the progress in treatment, in-hospital stroke is still tragic. Unlike community-onset stroke, the incidence of in-hospital stroke has not been investigated in detail. More than 30,000 patients are estimated to have an in-hospital stroke every year in the United States, and approximately 7% of all stroke events occur in the hospital [7,8]. More than 90% of all stroke events in the hospital were ischemic strokes [8]. Patients with in-hospital stroke have more severe disabilities than those with community-onset stroke due to their comorbidities [9,10]. In addition, they have fewer opportunities to receive acute therapy, especially IV thrombolysis [8,11,12]. One reasonable explanation is that patients with in-hospital stroke are often ineligible for IV thrombolysis due to their various situations: they have undergone surgery or a procedure, have received complicated antithrombotic therapy, or have fatal comorbidities, such as intracranial hemorrhage, trauma, and gastric bleeding. However, unfortunately, delayed recognition of in-hospital stroke may be crucial.

Although hospitalized patients are basically monitored and observed by medical staff, the time from symptom onset to starting treatment is relatively more prolonged in patients with in-hospital stroke than in community-onset ischemic stroke [13]. Moreover, inpatient populations are often at risk of missing their opportunity for reperfusion therapies such as IV thrombolysis and EVT. A prospective cohort study in Canada demonstrated that inpatients waited 4.5 h from stroke onset to first neuroimaging, whereas outpatients waited for only 1.2 h [13]. It has also been shown that patients with in-hospital ischemic stroke may experience more severe neurological outcomes than patients with community-onset ischemic stroke [14]. As we know, rapid response is the most important factor in saving the patient’s brain from stroke, but the real situation of in-hospital stroke is far from “time is brain”.

One of the best ways to solve the problem is education for medical staff. In fact, more than 60% of in-hospital strokes are initially recognized by nurses [10], and neurologists are not the first witnesses. Lack of knowledge and delayed recognition of the nonexperts may contribute to the prolonged duration from the onset of treatment [14]. Therefore, to achieve a prompt response to stroke, all medical staff should understand the risks and characteristics of in-hospital stroke. Illustrating the real situation in the epicenter of in-hospital stroke would be helpful for all medical staff to prepare for in-hospital stroke.

Additionally, critical illness is regarded as having a strong association with developing stroke [15]. A previous study illustrated that the incidence of newly-onset stroke in the non-neurological intensive care unit (ICU) was 1.2%, which was quite higher than in the general population [16]. Therefore, when thinking about in-hospital stroke, it is quite important to scrutinize the causes and risks of stroke in the ICU. Similarly, understanding the latest knowledge about acute therapy for such patients with a high risk of in-hospital stroke would also be beneficial to shorten the duration from the onset to treatment.

However, stroke in the ICU has received limited investigation in the medical literature. To enhance our management of in-hospital stroke, it is crucial to comprehend the clinical features and risks of stroke in the ICU. Early intervention for AIS can mitigate brain damage if we are able to recognize stroke in the ICU promptly. Thus, a review of the relevant literature is presented, and the real situation of stroke in the ICU is clarified.

## 2. Causes of Stroke in the ICU

### 2.1. Cardiovascular Diseases and Cardiac Surgery/Procedures

- Among the patients in the ICU, 9–25% would suffer from new-onset atrial fibrillation (AF) due to various situations.
- Stroke is a common thromboembolic complication due to infective endocarditis (IE). IV thrombolysis is not applicable, so EVT is expected to be beneficial.
- Perioperative stroke after coronary artery bypass graft (CABG) is caused by arrhythmias and hemodynamic instability.
- In thoracic endovascular aneurysm repair (TEVAR), more proximal stent landing and stent covering the left subclavian artery are the risks for perioperative stroke.
- After transcatheter aortic valve implantation (TAVI), silent infarction could be detected in 70% of the patients, and 1.1–6.7% would suffer from symptomatic stroke.

An observational, retrospective study in Korea demonstrated that 60% of in-hospital strokes were associated with surgery or procedures, and the most common cause of in-hospital stroke was cardiac surgery [17]. Commonly, patients with cardiovascular disease undergoing surgery or critical illness are at increased risk of stroke and are often admitted to the ICU. Only a few reports have described in-hospital stroke patients’ characteristics in detail. Approximately 0.2% of patients who underwent cardiac surgery developed ischemic stroke during their hospitalization, and patients in cardiology-related departments were at 10-fold higher risk for stroke than those in other departments [17]. Another study showed that approximately 40% of patients with in-hospital stroke were hospitalized due to cardiovascular diseases [18]. Naturally, cardiovascular diseases and cardiac surgery or procedures are strongly related to in-hospital stroke; therefore, it is imperative to pay close attention to patients with cardiovascular diseases during hospitalization.

AF is a well-known etiology of stroke. Patients sometimes develop new-onset AF during their hospitalization and may have worse outcomes if they develop a stroke than patients without AF [19]. In particular, 9–25% of patients who were admitted to an ICU presented with new-onset AF triggered by clinical illness, such as surgery and sepsis [20,21]. Cardiac surgery is the most responsible, but 10–20% of patients who underwent noncardiac surgery experienced perioperative AF [22,23]. Inflammation, sympathetic response, and cardiac hypoperfusion induced by surgery may synergistically contribute to AF [23]. Similarly, AF is triggered in sepsis by bacterial infection, oxidative stress, electrolyte imbalance, and vasopressor agents [24,25]. A systematic review reported that 13.5% of patients with sepsis had new-onset AF, and increased mortality in the ICU was found on multivariable metaregression (RR: 2.12, 95% CI: 1.86–2.43) [24]. However, the stroke risk of new-onset AF due to critical illness may vary for each patient, and routine anticoagulant agent use is still debated because of the lack of sufficient evidence [26]. Therefore, stroke risk should be assessed individually.

IE is a devastating infectious disease. *Staphylococcus aureus* is the most common cause of IE, and nearly half of IE cases are associated with surgery [27]. This fatal bloodstream infection is strongly associated with systemic embolism by vegetation formed on the mitral valve. Ischemic stroke associated with IE accounts for over 60% of all embolic events of IE, and the most affected artery is the middle cerebral artery [27,28]. *Staphylococcus aureus*, *Candida*, *Haemophilus species*, *Aggregatibacter species*, *Cardiobacterium hominis*, *Eikenella corrodens*, and *Kingella species* are regarded as the main causes of ischemic stroke induced by IE [27]. Regrettably, even though large vessel occlusion occurs with IE, IV thrombolysis is typically not applicable for patients with ischemic stroke induced by IE since the presence of IE itself and an abnormal coagulation profile are listed in the exclusion criteria for IV thrombolysis [29], whereas the efficiency of EVT has been reported recently [30,31]. A systematic review of the literature showed that 37% of the patients had better outcomes 90 days after EVT [31]. In fact, 13.3% of all cases experienced intracranial hemorrhage after EVT in the study, but we should not give up thinking about how to rescue IE patients from ischemic stroke.

Generally, cardiac surgical procedures, such as graft interposition of the ascending aorta, CABG, percutaneous coronary intervention (PCI), and implantation or removal of external pacemakers, contribute to perioperative stroke. CABG is one of the most common cardiac surgeries. The incidence of stroke after CABG is about 1%, and the incidence is gradually decreasing over the decades [32,33]. Stroke commonly occurs two days after the surgery due to arrhythmias and hemodynamic instability [32,34]. Age and several comorbidities, including severe kidney disease, severe chronic lung disease, carotid artery disease, and other heart diseases, are regarded as crucial risk factors for stroke after CABG [32,35,36,37]. Off-pump coronary artery bypass (OPCAB), which is performed without cardiopulmonary bypass, is associated with a reduced risk of neurological complications compared to on-pump CABG [38,39]. Compared to CABG, PCI is associated with a relatively lower risk of procedural stroke [40], less than 0.5% [41]. Stroke after PCI occurs commonly within 48 h [32,42]. Proper anticoagulation and operator experience are the keys to preventing stroke after PCI [32].

TEVAR is one of the common vascular surgeries. Approximately 70% of thromboembolic events result from aortic manipulation and scraping atheromatous plaque; the rest are associated with hypoperfusion during surgery [43]. The stroke rate after TEVAR is 2.9–11.5% [44,45,46], and age, body mass index, diabetes mellitus, chronic obstructive pulmonary disease, urgency of repair, and type of anesthesia are recognized as significant contributors [45]. In addition, more proximal stent landing and stent covering the left subclavian artery are considered to be associated with stroke [44,47].

TAVI is an alternative procedure to surgical aortic valve replacement or severe aortic valve stenosis and has emerged worldwide in recent years. By a catheter procedure, the bioprosthetic valve is delivered and placed on the aortic valve, and TAVI now prevails worldwide because of its less invasiveness. On the other hand, embolic stroke after TAVI is an important problem. During the aortic valve replacement procedure, embolic debris from the valve and aorta can scatter and cause ischemic stroke [48,49]. Regardless of whether the transfemoral approach or transapical approach is used, new silent cerebral infarctions detected by MRI were found in approximately 70% of patients after TAVI [50,51,52]. Further, several studies showed that 1.1–6.7% of patients who underwent TAVI experienced a symptomatic ischemic stroke or transient ischemic attack within 30 days after the procedure [53,54]. Cerebral embolic protection devices (CEPDs) are expected to reduce debris and embolic complication. In the procedure, CEPD is delivered by catheter and deployed in the aortic arch. CEPD may capture the debris or deflect the debris away from cerebral arteries. Although CEPD use seems to be reasonable, the efficiency has not been proven, and further study is needed [55].

### 2.2. Extracorporeal Circulation and Left Ventricular Assist Devices (LVADs)

- Of the patients maintained with extracorporeal membrane oxygenation (VA-ECMO), 1.4% experience stroke. EVT is expected to be an effective treatment for AIS during ECMO.
- Approximately 4% of the patients who were supported by Impella would experience a stroke.
- Among the patients who were implanted with left ventricular assist devices (LVADs), 14% would suffer from stroke within a year after the implantation.

In a case with cardiogenic shock, the use of extracorporeal circulation, including intra-aortic balloon pumping (IABP), VA-ECMO, and Impella (Abiomed Inc., Danvers, MA, USA), an intravascular heart pump device, are crucial for providing life support. IABP is used worldwide in the ICU to support left ventricular function. According to the Intra-aortic Balloon Pump in Cardiogenic Shock (IABP-SHOCK) II trial, a multicenter, open-label, randomized study, there was no significant relationship between IABP and stroke [56,57].

VA-ECMO is another life-supporting device used in cases with refractory lung and heart failure, and its use has increased in this decade [58]. Neurological complications are strongly associated with death or long-term disability, but their frequency has not been described in detail [59]. Cho et al. investigated 15,872 patients who were supported by VA-ECMO. Of these patients, 215 (1.4%) experienced an ischemic stroke, and they found that acidemia, hypoxemia, and abnormal coagulation status were independent predictors of acute brain injury [60]. In addition, microemboli in cannulae and pulseless flow are regarded as other risk factors [61]. Cardiac arrest or global ischemia may lead to loss of cerebral autoregulation [62], and the patients become more vulnerable to the up/downregulation of cerebral circulation.

The Impella is a new percutaneous left ventricular assist device for cardiogenic shock. It is located at the left ventricle and pumps blood to the aorta to reduce the workload on the left ventricle [63]. The incidence of ischemic stroke associated with its use is unknown. One previous single-center study in the United States showed that 3 of 79 patients (3.8%) in whom the Impella was used developed ischemic stroke. Of them, two patients were maintained with inadequate anticoagulation [63]. The Impella and IABP are often combined with ECMO, making it difficult to assess the risk of stroke accurately. However, proper management during extracorporeal circulation, including appropriate anticoagulation, monitoring of cannulae, and optimizing oxygenation, is crucial to prevent stroke.

LVADs are mechanical pumps that support the circulatory system. They are implanted in patients waiting for a heart transplant. It has been reported that the rate of ischemic stroke events within 30 days after the implantation of LVADs was 0.06–0.62 per patient-year [64,65,66,67,68]. Ischemic stroke during the peri-implantation period might be associated with hypercoagulation, AF, and infection due to surgery [69,70,71]. After that period, continuous abnormal coagulation, inflammation, and shear stress caused by LAVDs are responsible for ischemic stroke. In addition, patients with LVADs must be maintained on anticoagulant agents [72,73,74]. However, adequate management is sometimes challenging to control hemorrhagic or thromboembolic event risks. One multicenter observational study showed that 14% of patients with LVADs experienced stroke one year after implantation [74]. Stroke is a fatal complication for patients with LVADs, who may lose their chance to undergo heart transplantation. Therefore, we should pay close attention to patients with LVADs.

It is difficult for patients maintained by extracorporeal circulation devices to receive acute therapy for AIS, and they are on anticoagulant agents, so IV thrombolysis is not applicable. Though EVT may be theoretically possible, it is also quite challenging because of the choice of vascular access. However, there are some case series of patients treated with EVT during ECMO [75]. In addition, successful EVT for patients with LVADs has been reported [68]. Further study is needed to determine the efficacy of EVT for patients with extracorporeal circulation devices.

### 2.3. Noncardiac and Surgery/Procedure

- About one-fourth of the patients who undergo left upper lobectomy suffer from perioperative stroke.
- The incidence of perioperative stroke treatment for an unruptured intracranial aneurysm is 6.1%. In addition, patients with subarachnoid hemorrhage have a great risk of delayed cerebral ischemia (DCI).

In general, perioperative stroke is less commonly associated with noncardiac surgery than cardiac surgery. Though it is still uncommon, the incidence of perioperative stroke following noncardiac surgery has been gradually increasing [76,77]. Smilowitz et al. investigated 10,581,621 hospitalizations for noncardiac surgery in the United States and reported that the incidence of perioperative stroke was 0.54% in 2004 and 0.78% in 2013 [76]. Furthermore, stroke after surgery is recognized as the crucial factor for mortality within 30 days [78,79], making it imperative for physicians to understand its importance.

Thoracic surgery and neurosurgery are said to have a higher incidence of stroke than general surgery, in this order [76]. The frequency of cerebral infarction after pulmonary resection is 0.2–0.8% [80,81,82], but recently, left upper lobectomy has been suggested to be a new threat [83]. Pulmonary vein thrombosis may occur in the long left superior pulmonary vein stump, resulting in cerebral infarction [84]. In a Japanese, multicenter, retrospective study, 610 patients who developed stroke following lobectomy and 773 patients without stroke were analyzed. It was found that 71 of 122 patients (25.3%) who underwent left superior lobectomy developed stroke after the surgery, and subgroup analysis showed that left upper lobectomy appeared to have a stronger relationship with perioperative stroke than other types of lobectomy [82]. However, since the mechanism and clinical features of perioperative stroke are not well understood, further research is necessary.

Surgical clipping and endovascular coiling are the standard therapy for intracranial aneurysms. There has been little investigation on stroke after surgical treatment for ruptured and unruptured intracranial aneurysms [85,86,87]. In a previous cross-sectional study, the incidence of perioperative stroke after treatment for unruptured intracranial aneurysms was 6.9% (4.3% for surgical clipping, 8.1% for endovascular coiling, and 1.9% for assisted coiling with stent or balloon) [88]. That study also showed that assisted coiling by stent or balloon might prevent perioperative stroke compared to surgical clipping alone. Otherwise, the size of the aneurysm and the location and history of stroke were supposed to be risks for neurological complications after unruptured intracranial aneurysms [89]. Concerning ruptured intracranial aneurysms and subarachnoid hemorrhage, the severity of subarachnoid hemorrhage seems to be associated with perioperative stroke after surgical clipping [87]. Vasospasm caused by subarachnoid hemorrhage is a well-known phenomenon that is closely related to DCI. The Fisher scale and modified Fisher scale are well-known scoring systems for predicting DCI based on radiological findings and evaluating the thickness and location of hemorrhage [90]. The subarachnoid hemorrhage early brain edema score (SEBES) is another predictive model for DCI that assesses the radiological changes at the sulci and gray-white matter junction caused by brain edema [91]. Proper assessment of the risk of DCI is critical for preventing neurological complications after subarachnoid hemorrhage.

### 2.4. Sepsis and Stroke

Up to 80% of patients with bacteremia may experience cardiovascular events during their hospitalization [92,93], and younger patients seem to have an increased risk of stroke after sepsis [93]. Sepsis and severe infection lead to stroke through several mechanisms. Sepsis per se may drive AF, which is a major cause of stroke, as mentioned above. Systemic inflammation, electrolyte abnormalities, and organ dysfunction due to sepsis can cause AF, and the incidence of AF induced by sepsis is reported to be 1.9–25.9% [94]. In a retrospective, population-based cohort study in California, 3,144,787 hospitalized patients were investigated, and 2.6% of severe sepsis patients with new-onset AF developed in-hospital stroke and had significantly higher in-hospital mortality than those without AF [95].

In addition, sepsis often provokes disseminated intravascular coagulation. Via the extreme hypercoagulopathy driven by the systemic inflammatory response syndrome, a thrombus may be formed in numerous arteries and veins, resulting in ischemic stroke [96]. Shao et al. established a predictive model for stroke after sepsis that consisted of valvular heart diseases, heart failure, coagulopathy, lymphoma, peripheral vascular diseases, pulmonary circulation disorders, and renal failure [97]. Unfortunately, the study was not able to demonstrate high predictive accuracy (maximum area under the receiver-operating characteristic curve 0.58) due to its low incidence. However, their findings showed that the combination of patient conditions associated with sepsis might contribute to the development of stroke after sepsis.

### 2.5. Medications Associated with Stroke

- We have to pay a lot of tension when the patients receiving antithrombotic agents undergo surgery.
- Some medications are regarded as the cause of stroke, so close observation is needed when those are administrated.

Stopping antithrombotic agents or inadequate antithrombic therapy are significant risks for stroke. When a patient who has a higher risk for bleeding undergoes surgery or a procedure, we sometimes stop antithrombotic therapy several days before surgery or a procedure to avoid hemorrhagic complications associated with an operation. The timing of stopping and restarting the treatments is suggested by several guidelines on the bases of the type of antithrombotic agents and the invasiveness of the surgery [98]. In addition, we can assess the individual risk for cardiac embolism due to AF by the CHADS_2_ score or CHA_2_DS_2_-VASc score [99]. To avoid thrombotic events, we have to pursue the optimal timing to stop and start antithrombotic agents during the operation.

Some medications include the risk of stroke as an adverse effect. Morto et al. conducted a literature review of randomized, controlled trials of medications associated with stroke and reported the list of high-risk medications [100]. According to that report, erythropoietin- and erythropoiesis-stimulating agents, contraceptives or hormone replacement therapies, bevacizumab, tamoxifen, and antipsychotics were categorized as medications with a high level of evidence for their association with stroke. In addition, ponatinib, nilotinib, darunavir, and hormonal agents were identified as medications with a moderate level of evidence for the association.

Erythropoietin- and erythropoiesis-stimulating agents are the treatment for anemia in chronic kidney disease, and they increase the hemoglobin level. In general, chronic kidney disease is a major comorbidity of critically ill patients, so these agents are often used in the ICU. It is thought that a higher hemoglobin level in chronic kidney disease results in hypertension and hyperviscosity, which then predisposes to stroke, and the risk of stroke would increase along with the target hemoglobin levels [100,101]. Thus, close monitoring is required to prevent stroke events.

Antipsychotics, especially quetiapine, are used in the ICU for psychiatric symptoms. Many factors surrounding ICU patients, such as severe illness, mechanical ventilation, drugs, and environment, contribute to the development of delirium, which is critically related to hospital mortality [102]. Therefore, to prevent delirium in the ICU, antipsychotic treatment is essential. The efficiency of quetiapine in the ICU has been proven by several studies [103,104,105], so it is widely used for critically ill patients. On the other hand, several studies demonstrated the association between antipsychotics and stroke [106]. The Danish nationwide study demonstrated that continuous low-dose quetiapine use increased the risk of major adverse cardiovascular events and ischemic stroke (aHR 1.52, 95%CI: 1.35–1.70, *p* < 0.001; aHR 1.37, 95% CI: 1.13–1.68, *p* = 0.002) [107]. The biological mechanism of how antipsychotics cause stroke has not been elucidated, but it is suggested that antipsychotics are associated with thromboembolism [108,109].

## 3. Stroke Mimics in the ICU

- Upper limb weakness due to Todd’s paralysis is difficult to distinguish from stroke, but it is benign and reversible.
- Nonconvulsive status epilepticus (NCSE) with coma is characterized by prolonged loss of consciousness, and MRI and electroencephalogram are helpful for diagnosis.
- Hypoglycemic hemiparesis is a rare complication of severe hypoglycemia. It is promptly reversed by glucose administration.

Stroke may bring various symptoms, such as limb weakness, facial palsy, numbness, loss of vision, dysarthria, aphasia, and disturbance of consciousness. Such neurological deficits with sudden onset are mainly characterized by stroke. However, those manifestations also result from other diseases: “stroke mimics”. It is described that the frequency of stroke mimics in the emergency department might account for up to 25% of the patients with suspected stroke [110]. Therefore, understanding how other diseases mimic stroke is essential.

Seizure is a common neurological disorder in the ICU, and sometimes, postictal Todd’s paralysis is challenging to distinguish from stroke [111]. Todd’s paralysis is the reversible weakness or paralysis after ictal discharge, and the mechanism is not unveiled clearly. The frequency ranges from 0.6 to 13.4% [112], and the upper limb may be affected commonly [113]. The duration of Todd’s paralysis ranges from a half hour to more than a day, based on the severity of the brain injury [114]. Although it is difficult to distinguish Todd’s paralysis from stroke by clinical manifestations, this condition is benign and reversible. Therefore, the assessment for stroke is prioritized when we notice the patient’s weakness. Moreover, NCSE, which is a type of status epilepsy without an obvious motor activity, mimics a stroke. NCSE may usually occur after a generalized seizure, and, especially, NCSE with coma, which is characterized by prolonged loss of consciousness, often needs intensive care [115]. It is difficult to distinguish NCSE with coma from stroke by clinical manifestation, but MRI testing and electroencephalogram would be helpful.

Severe hypoglycemia is often experienced in the ICU and may cause focal neurological deficits. Particularly, hypoglycemic hemiparesis, which is a rare manifestation of hypoglycemia, masquerades as stroke [116]. A literature review described that the average blood glucose level of the patients with hypoglycemic hemiparesis was 1.8 mmol/L, and the right limbs were affected more than the left limbs [117]. Additionally, diffusion weight imaging hyperintensity in the internal capsule appears in patients with hypoglycemic hemiparesis, similar to AIS [118]. However, hypoglycemic hemiparesis could be recovered by treatment with glucose. Therefore, the first step of the AIS treatment is to test blood glucose levels and promptly administer to patients with hypoglycemia.

## 4. Education for Medical Staff

As we demonstrated, patients in the ICU have numerous risks for in-hospital stroke. Whereas the response protocol for community-onset stroke has been discussed, that for stroke in the ICU is scarcely investigated [8,119]. Because of the heterogeneity and complexity surrounding the patients in the ICU, establishing a uniform strategy for AIS in the ICU is quite difficult. Even though patients suffer from minor strokes that would not need acute therapy, antiplatelet treatment is quite important to prevent the recurrence of in-hospital stroke [120,121]. As a first step, providing regular education and feedback to nonexperts can be an effective way to improve their daily practice [8].

## 5. Conclusions

Representative issues related to stroke in the ICU were reviewed. Patients admitted to the ICU have diverse situations, and their risks of stroke vary greatly. In particular, patients who undergo surgery or procedures are always at risk for stroke. In addition, stroke can be a fatal complication in patients with critical illnesses. Therefore, it is not feasible to consider in-hospital stroke separate from the ICU setting. Further investigation is needed to fully understand the current state of stroke in the ICU and establish better clinical practices.

## Data Availability

Not applicable.
